# A Framework for Identification of Healthy Potted Seedlings in Automatic Transplanting System Using Computer Vision

**DOI:** 10.3389/fpls.2021.691753

**Published:** 2021-07-30

**Authors:** Xin Jin, Chenglin Wang, Kaikang Chen, Jiangtao Ji, Suchwen Liu, Yawei Wang

**Affiliations:** ^1^Department of Agricultural Machinery, Henan University of Science and Technology, Luoyang, China; ^2^Department of Robotics Engineering, Chongqing University of Arts and Sciences, Yongchuan, China; ^3^Design and Simulation Unit, Collaborative Innovation Center of Machinery Equipment Advanced Manufacturing of Henan Province, Luoyang, China

**Keywords:** seedling identification, computer vision, identification algorithm, vision, automatic transplanting

## Abstract

Automatic transplanting of seedlings is of great significance to vegetable cultivation factories. Accurate and efficient identification of healthy seedlings is the fundamental process of automatic transplanting. This study proposed a computer vision-based identification framework of healthy seedlings. Vegetable seedlings were planted in trays in the form of potted seedlings. Two-color index operators were proposed for image preprocessing of potted seedlings. An optimal thresholding method based on the genetic algorithm and the three-dimensional block-matching algorithm (BM3D) was developed to denoise and segment the image of potted seedlings. The leaf area of the potted seedling was measured by machine vision technology to detect the growing status and position information of the potted seedling. Therefore, a smart identification framework of healthy vegetable seedlings (SIHVS) was constructed to identify healthy potted seedlings. By comparing the identification accuracy of 273 potted seedlings images, the identification accuracy of the proposed method is 94.33%, which is higher than 89.37% obtained by the comparison method.

## Introduction

Vegetables are rich in vitamins, minerals, and crude fiber, which are indispensable food for humans. Vegetable production is an important production activity of normal supply, and its efficiency directly affects vegetable yield. The introduction of industrial production technology into vegetable factories can greatly improve the efficiency of vegetable production.

In the production process of a vegetable factory, vegetable seeds are usually cultured in pots after being washed and soaked. The matrix placed in the pot can be used to nourish the seed growth. Potted seedlings are placed in trays for cultivation. Until the potted seedlings grow to meet the standard of healthy seedlings, the potted seedlings will be transplanted for the preparation of subsequent vegetable production. Therefore, the accuracy and efficiency of potted seedling transplanting is an important process of vegetable production.

The automatic transplanting of healthy potted seedlings can not only ensure the integrity of seedling growth but also reduce the time consumption of manual operation in the whole process of vegetable factory production. Many automatic transplanting systems have been developed for transplanting different kinds of potted seedlings (Han et al., 2018a,b; Rahul et al., 2019; Jin et al., 2020). The transplanting mechanism was designed and the theory of automatic transplanting was analyzed in literature (Sun et al., 2017; Vivek et al., 2017). A mini-automatic transplanting machine was developed, and the performance of its control system was investigated by Yang et al. (2020). The above reports mainly conducted the transplanting speed and success rate under the mechanical structure condition.

Vision-based automatic transplanting can distinguish healthy potted seedlings from unhealthy ones, which may promote an accurate rate of transplanting potted seedlings. The visual identification of the growth state of potted seedlings plays a vital role in the automatic transplanting of healthy potted seedlings. The accurate visual identification of healthy potted seedlings is the first step to ensure the seedlings transplanting without damage. The development of a robust identification algorithm for healthy potted seedlings can make a vision-based transplanting system play its advantages in vegetable factory production.

Machine vision technology has been applied to multiple fields (John, 2017; Mauro et al., 2018; Wang et al., 2018; Kim et al., 2020). For the detection of seedling growth status, an early method could be found in Ling and Ruzhitsky (1996), which could measure tomato seedling canopy with an adaptive threshold algorithm and the Otsu method. Lin et al. estimated the leaf area of seedlings by the projected contour image and proposed an image processing method based on elliptic Hough transform to determine the overlapping position of seedlings leaves (Lin et al., 2002). A model for estimating seedling leaf area was developed using vision technology in Karimi (2009), where the model used the linear regression equation of leaf length and width obtained by a vision to estimate the leaf area. Tong et al. combined the region center of cross-border leaves with the improved watershed segmentation method to measure the leaf area and then estimated the quality of vegetable seedlings through the leaf area (Tong et al., 2013). A linear structured light vision system was designed to measure seedling surface information as described in Feng et al. (2013). In the system, one color image of the seedling line with linear structured light was used to measure the seedling height, and the other color image of the seedling line without linear structured light was used to identify the size of the seedling leaf, a color index of 2G-R-B was used to distinguish the seedling leaf from the substrate, and the Otsu dynamic threshold was adopted to extract the leaf area. Ashraf et al. (2014) used the theory of machine vision to inspect seedlings for sorting and inspecting grafted seedlings. An automated corn seedling phenotyping platform based on a time-of-flight (TOF) camera and an industrial robot arm was developed by Hang et al. (2017). Their method used the TOF camera to obtain the data of three-dimensional (3D) point cloud of seedlings, then used a 3D-to-2D projection and an x-axis pixel density distribution algorithm to segment and match the corn seedlings (Hang et al., 2017). A comprehensive image processing flow, which used discontinuous gray to segment the leaf area of seedling, applied an order statistic filtering to reduce the random noise and utilized the Otsu algorithm to segment the seedling image, was presented to extract the feature information of graft seedlings in Zhang et al. (2015). Franck et al. proposed a fast 3D reconstruction method for seedling phenotyping and measured the seedling surface features by using the developed computer vision technology (Golbach et al., 2016). A method based on the photometric stereo for measuring the seedling leaf morphology was reported in Feng et al. (2018). Yang et al. combined filtering and clustering segmentation algorithms to process the 3D point cloud data of overhead view of seedling using the imaging principle of RGB-D camera (Yang et al., 2019).

Different from the reported methods for detecting seedlings, this study proposed a new framework for identifying healthy seedlings based on the physical transplanting prototype developed by a research group using computer vision technology. The purpose of this study is to identify healthy seedlings quickly and accurately for an automatic seedling transplanting system.

## Materials and Methods

### System Framework

**Figure 1:**
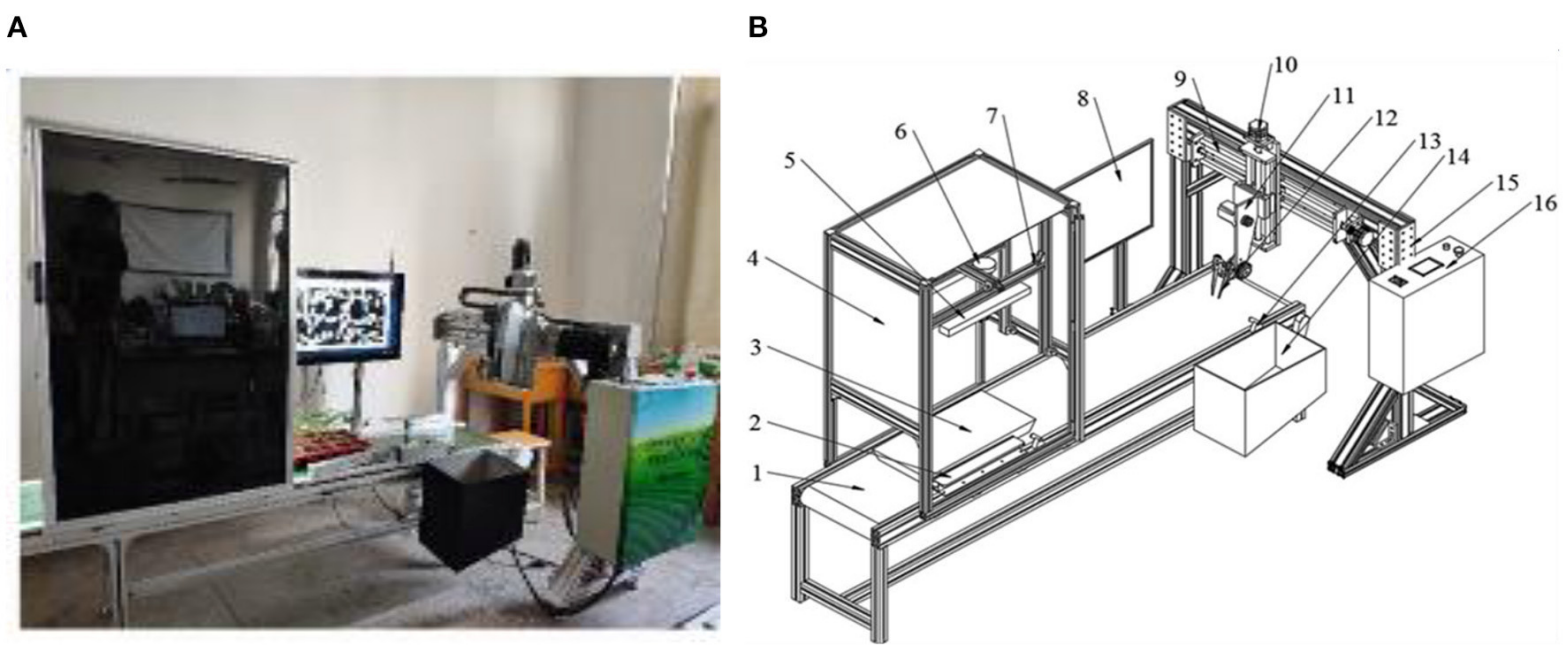
System modeling. **(A)** Physical prototype; **(B)** System structure diagram: 1, conveyor belt; 2, limit device; 3, tray with seedlings; 4, lightbox; 5, compensatory light; 6, charge-coupled device (CCD) camera; 7, frame; 8, personal computer; 9, cross slide; 10, stepping motor; 11, manipulator mounting plate; 12, end-effector; 13, diffuse reflection laser sensor; 14, recovery box; 15, gantry; 16, programmable logic controller (PLC).

The physical prototype of automatic seedling transplanting is shown in Figure 1A and its specific structure is shown in Figure 1B, which consists of a conveyor unit, a visual detection unit, and a transplanting unit.

Seedlings are planted in trays in the form of pot seedlings. The conveyor unit is mainly responsible for the position movement of tray seedlings, which is composed of a conveyor belt, a limit device, and a frame. The conveyor belt is mounted on the frame and driven by a stepper motor. The visual detection unit is mounted above the conveyor belt. To ensure that the visual detection unit can effectively collect image information of potted seedlings, the conveyor belt is equipped with a guide rail and a guide wheel. When the tray passes through the guide rail area, the horizontal placement posture of the tray will be automatically corrected. The edge line of the tray and the edge line of the conveyor belt will be parallel after the correction. The end of the conveyor belt is equipped with a recovery box of potted seedlings, which is used for the removal and recovery of seedlings of poor quality.

The image of tray seedlings was obtained by a charge-coupled device (CCD) camera with a resolution of 1,280 ^*^ 960 (model MV-VD120SC, supplied by Micro-vision company, Xi'an in China). The distance between the camera and the conveyor belt is 700 mm. The CCD camera is connected to a personal computer (PC) by a cable with one USB3.0 interface. The images of tray seedlings obtained by the CCD camera were stored in the PC, which has 8 GB RAM, an Intel Core i5-4590 CPU, and a Windows 7 operating system. The software system of the image processing system running on the PC is OpenCV 3.0 and Matlab 8.3. The transplanting unit, including a gantry and an end-effector, is mainly to carry out the sorting operation of potted seedlings. A manipulator moves on a gantry according to the seedling information, and an end-effector equipped at the end of the manipulator is applied to grasp the target seedling. The actions of the gantry and the manipulator are controlled by a programmable logic controller (PLC).

**Figure 2:**
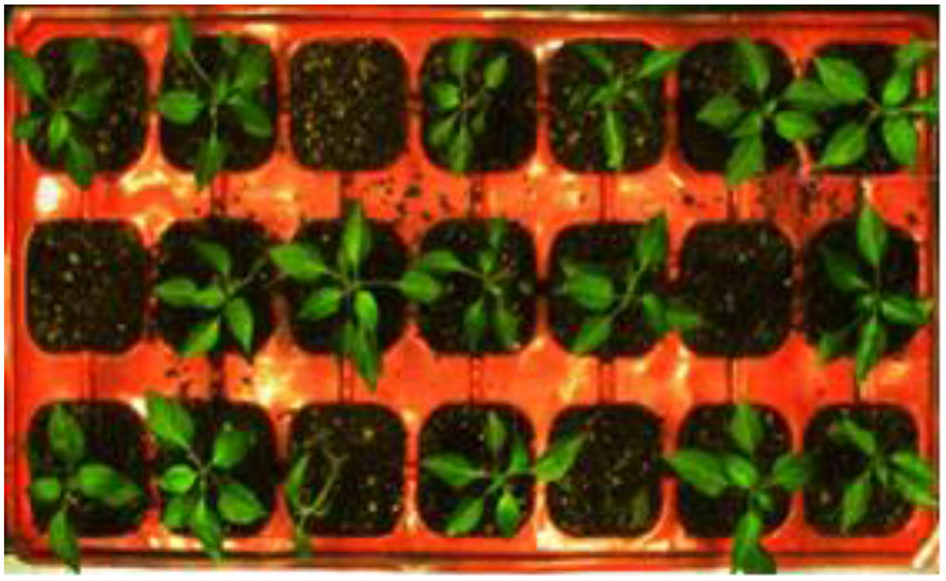
The original tray image with potted seedlings.

When the tray is transported to the visual detection system, the belt stopped for 1 s and the visual detection unit is triggered to acquire the image information of tray seedlings as shown in Figure 2. The visual detection of the growth status of seedlings is carried out, where the seedling detection algorithm will be specified in the following section.

### Seedling Image Preprocessing

The surface color of the potted seedlings is an important factor to reflect its growth status; however, the acquired image of potted seedling includes noises due to various external interferences. There are different objects in the image of tray seedlings, which will increase the difficulty of identifying healthy seedlings.

The potted seedlings are green with different surface colors from that of the seedling matrix and the tray. The seedling matrix is brown-black. The tray is in red color. Therefore, image preprocessing is necessary and a proper color index needs to be developed for the color image of potted seedling in the *RGB* color space.

Two-color indexes are proposed as shown in Equations (1) and (2) to calculate the color differences of all pixels in the images so that the green and red color components can be enhanced, and the rest of the components can be weakened in the seedling image.

(1) $$\begin{array}{l} TG=\left(G\times 3-R-B\right)/3 \end{array}$$

(2) $$\begin{array}{l} TR=\left(R\times 3-G-B\right)/3 \end{array}$$

where *TG* and *TR* are the green color component and the red color component after image preprocessing, respectively. *R, G*, and *B* are color components of the pixels in the *RGB* color space of the original image.

To investigate the surface information of seedlings more comprehensively, an index of the grayscale image preprocessing is proposed as shown in Equation (3).

(3) $$\begin{array}{l} Y=R\times 0.2989+G\times 0.5870+B\times 0.1141 \end{array}$$

where *Y* is the gray value of the pixel in the grayscale image and *R, G*, and *B* are color components of the pixels in the *RGB* color space of the original image. Thus, the image preprocessing of potted seedlings is finished to prepare for the subsequent image segmentation.

### Segmentation and Denoising of Seedling Image

**Figure 3:**
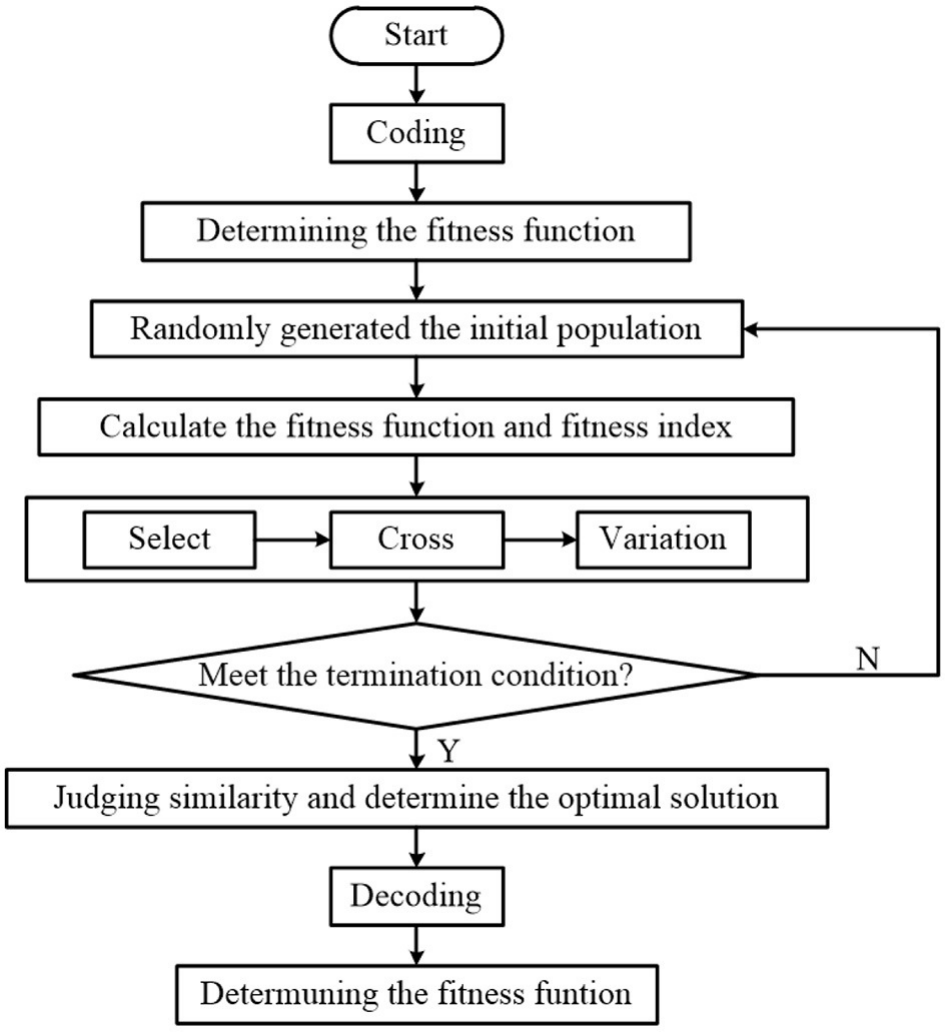
Flowchart of seedling image segmentation.

After the image preprocessing, an image segmentation algorithm is proposed using an optimal thresholding method based on the genetic algorithm. The algorithm flow is shown in Figure 3.

In the encoding, the grayscale image is binary coded to produce a 16-bit binary number. The first eight bits represent the segmentation threshold *T*_1_, and the remaining eight bits represent the segmentation threshold *T*_2_. The number of seedlings affects the fitness calculation of each generation. The initial generation quantity should be set reasonably. After a lot of simulation, the number of pepper seedlings is set to 21, and the maximum number of breeding algebras is set to 100.

- Decoding: The binary array generated by the encoding is decoded and converted to a value between 0 and 255 for the fitness value.
- Fitness function: Equation (4) is used as a function of fitness value, and the linear scaling of the fitness function is taken as follows:

(4) $$\begin{array}{r} F\left(T_{1},T_{2}\right)=-\overset{T_{1}}{\underset{i=0}{\sum }}\frac{P_{i}}{P_{C_{1}}}ln\frac{P_{i}}{P_{C_{1}}}-\overset{T_{2}}{\underset{i=T_{1+1}}{\sum }}\frac{P_{i}}{P_{C_{2}}}ln\frac{P_{i}}{P_{C_{2}}}\\ -\overset{l-1}{\underset{i=T_{2+1}}{\sum }}\frac{P_{i}}{P_{C_{3}}}ln\frac{P_{i}}{P_{C_{3}}} \end{array}$$

where *P*_*i*_ is the probability of occurrence of the *i*-th gradation. *T*_1_ and *T*_2_ are division thresholds with *T*_1_ < *T*_2_, which could divide grayscale images into *C*_0_, *C*_1_, and *C*_2_ categories. *P*_*C*0_, *P*_*C*1_, and *P*_*C*2_ represent the probability of occurrence of *C*_0_, *C*_1_, and *C*_2_, respectively.

- Choice: the roulette selection algorithm is applied.
- Cross: the main feature of the cross is the formation of two new individuals. The probability of crossover affects both the possibility of cross-operation and the speed of convergence. Therefore, it is especially important to choose the crossover probability. The crossover probability is set to 0.6.
- Variation: mutation is performed through simple mutation, and the mutation probability is set to 0.03.
- Termination criteria: when the algorithm performs to a predetermined algebra or the highest fitness value in the population is stable, the algorithm will end the operation.

A binary map of tray seedlings is generated by using Equation (5).

(5) $$\begin{array}{l} f\left(x,y\right)\{\begin{array}{cc} 1 & f\left(X,Y\right)\geq \\ 0 & otherwise \end{array}\text{ min}\left(T_{1}^{*},T_{2}^{*}\right) \end{array}$$

where $T_{1}^{*}$ and $T_{2}^{*}$ represent two thresholds obtained after the optimal threshold segmentation of the genetic algorithm 1 represents a seedling pixel, and 0 represents a background pixel. Then, the three-dimensional block-matching algorithm (BM3D) is applied to denoise the tray seedling image.

We use *I* to represent the image with noise and *P* to represent any matching block that has been divided. Set the block size of *P* to be *K* × *K*, and use *Q* to represent a sliding window block in the search process. When the size of the block is known, the pixel in the upper left corner of the image is used to represent the matching block. In the process of block matching, the appropriate step size “*h*” was first determined. The block is divided and searched according to the order from left to right and from top to bottom. The current block *P* is selected as the reference block, and the center point of *P* is considered as the reference point.

(6) $$\begin{array}{l} S\left(P\right)=\left\{Q\in I|d\left(X_{P},X_{Q}\right)|<\tau_{d}\right\} \end{array}$$

It is shown in Equation (6) that *S*(*P*) is a 3D matrix set including similar blocks. τ_*d*_ is the distance threshold in the search process. *d* is the distance between the matching blocks in the search process as shown in Equation (7).

(7) $$\begin{array}{l} d=h^{-1}\|X_{P}-X_{Q}\| \end{array}$$

where *X* is the matrix value of the matching block. Finally, the matrix block sets in order are arranged in order of magnitude. A 3D matrix of size *K* × *K* × *S*(*P*) is obtained. Then, the process of denoising in the 3D transform domain can be expressed by using the following equation:

(8) $$\begin{array}{l} F\left(P\right)=N_{3^{D}}^{-1}\left(\gamma \left(N_{3^{D}}\left(T_{S\left(P\right)}\right)\right)\right) \end{array}$$

where *N*_3*D*_ represents that *T*_*S*(*P*)_ makes 3D unitary transformation, and the arithmetic symbol is *N*_3*D*_. γ can be expressed as follows:

(9) $$\begin{array}{l} \gamma \left(x\right)=\{\begin{array}{c} 0,\;\left(|X|\leq \lambda_{3D}\sigma \right)\\ X,\;\left(|X|>\lambda_{3D}\sigma \right) \end{array} \end{array}$$

where λ_3*D*_ represents the threshold value of hard threshold filtering, and σ represents Gaussian white noise parameter.

When the filtering of the noise image is completed, each block will have an estimated value corresponding to it. *N*_*P*_ is used to represent the non-zero value in the matrix coefficient after filtering. *W*_*P*_. is used to represent the estimated value of the basic weight in the current block as shown in Equation (10).

(10) $$\begin{array}{l} W_{P}^{basic}\{\begin{array}{c} \frac{1}{N_{P}}\;(N_{P}\geq 1)\\ 1\;\left(N_{P}<1\right) \end{array} \end{array}$$

After calculating the basic estimation value of the 3D transform domain filtered by Equation (10), the final estimated weight can be obtained as shown in Equation (11).

(11) $$\begin{array}{l} W_{P}^{final}=\frac{{\left|\tau_{3D}\left(T_{S\left(P\right)}\right)\right|}^{2}}{{\left|\tau_{3D}\left(T_{S\left(P\right)}\right)\right|}^{2}+\sigma^{2}} \end{array}$$

It can be seen from Equation (11) that the larger the estimated weight is, the smaller the noise is in the real image. Finally, a real image could be obtained by calculating the average estimated weight of each overlapping block.

### Healthy Seedlings Identification

The leaf area “M” of the seedling is a visual representation of the seedling growth status. After segmenting the seedling image, a smart identification framework of healthy vegetable seedlings (SIHVS) is implemented for calculating the leaf area of each seedling. The SIHVS algorithm counts the number of pixels of the seedling leaf. The actual area of the seedling leaf can be obtained by proportional conversion with the leaf pixels. The regionprops function in MATLAB is used to calculate the area of each white region in the binary image. The parameter bws of the function regionprops calculates the number of pixels occupied by the white region in each region and could assign the calculating results to props. Then, the actual leaf area of each seedling is converted according to the relationship between the tray size and the pixels as shown in Equation (12).

(12) $$\begin{array}{l} M=K\times P \end{array}$$

where *M* is the actual leaf area of the seedling, *K* is a proportionality factor, which is the ratio of the actual size of the tray to the number of pixels in the tray, and *P* is the number of pixels occupied by the leaves of the seedling.

The ratio of the extracted leaf area value of the potted seedling to the hole area of the potted seedling is selected as the threshold “*F*” of leaf area. The SIHVS algorithm uses the threshold “*F*” to identify the healthy seedling. Healthy seedlings, sub-healthy seedlings, inferior seedlings, and empty cells could be identified and classified based on the average value, maximum value, and minimum value of the threshold *F*, which can be obtained by a lot of experiments. When confirming the growth state of a seedling, its threshold *F* is first calculated. If it is greater than the average value of *F*, it will be identified as a healthy seedling. If it falls between the average value and the minimum value of *F*, it will be identified as a sub-healthy seedling. If it is smaller than the minimum value of *F*, it will be considered as a seedling of poor quality. For empty cell recognition, the threshold *F* should be below 0.015 that could be confirmed by calculating the threshold value *F* of 100 empty cells. Thus, the healthy seedlings could be identified, and the details of the results will be given in the Results section.

## Results

### Segmentation and Denoising Results

**Figure 4:**
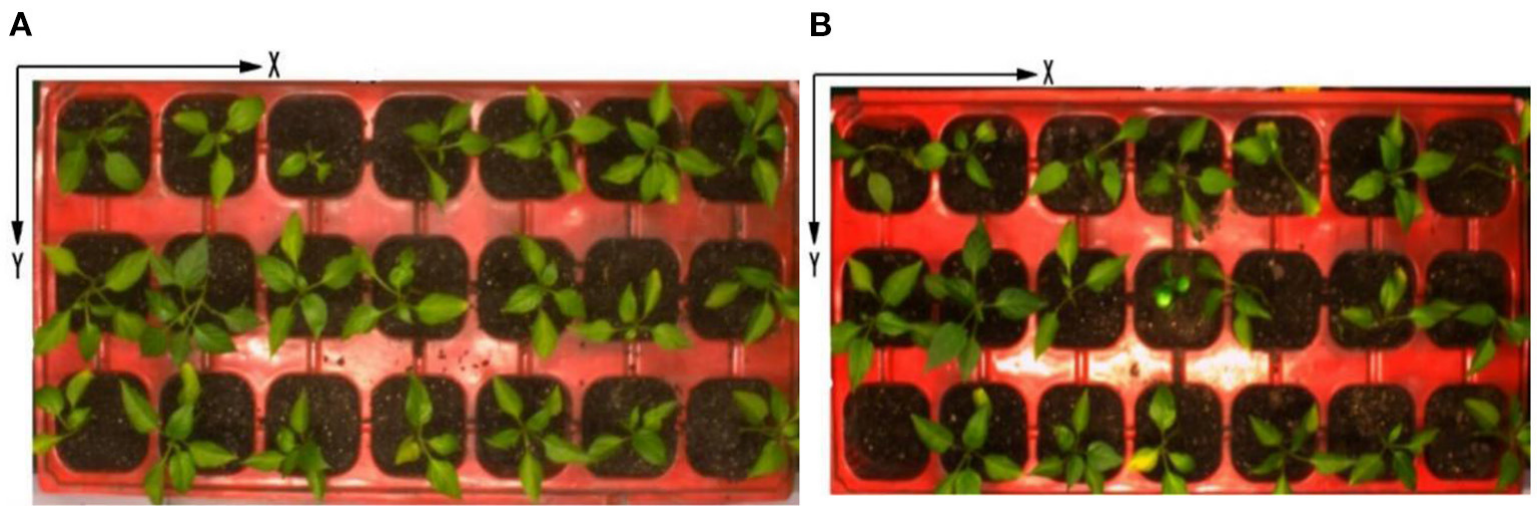
Original image of pepper seedlings.

**Figure 5:**
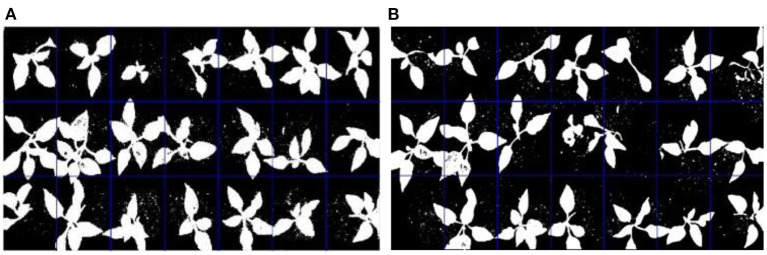
Threshold segmentation by the optimal threshold method.

**Figure 6:**
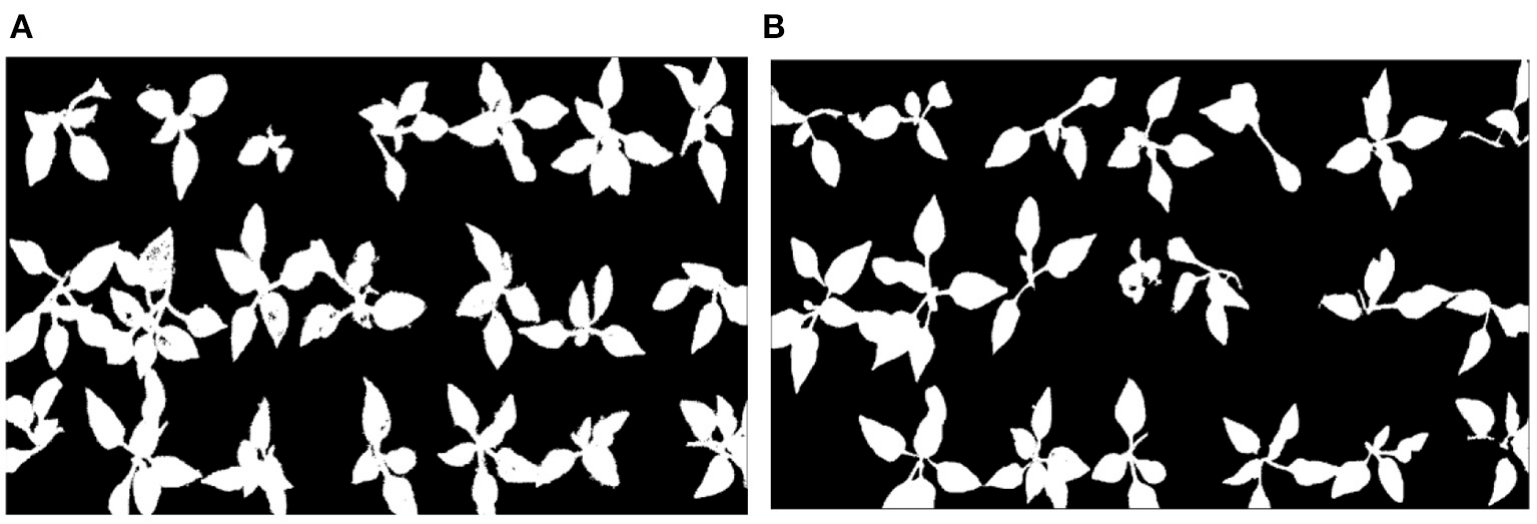
Image denoising by 3D block-matching algorithm (BM3D).

The original pictures of two trays of pepper seedlings are shown in Figures 4A,B. The segmentation results of the original pepper seedlings are shown in Figures 5A,B based on the optimal threshold method. Denoising results are shown in Figures 6A,B using the BM3D.

**Figure 7:**
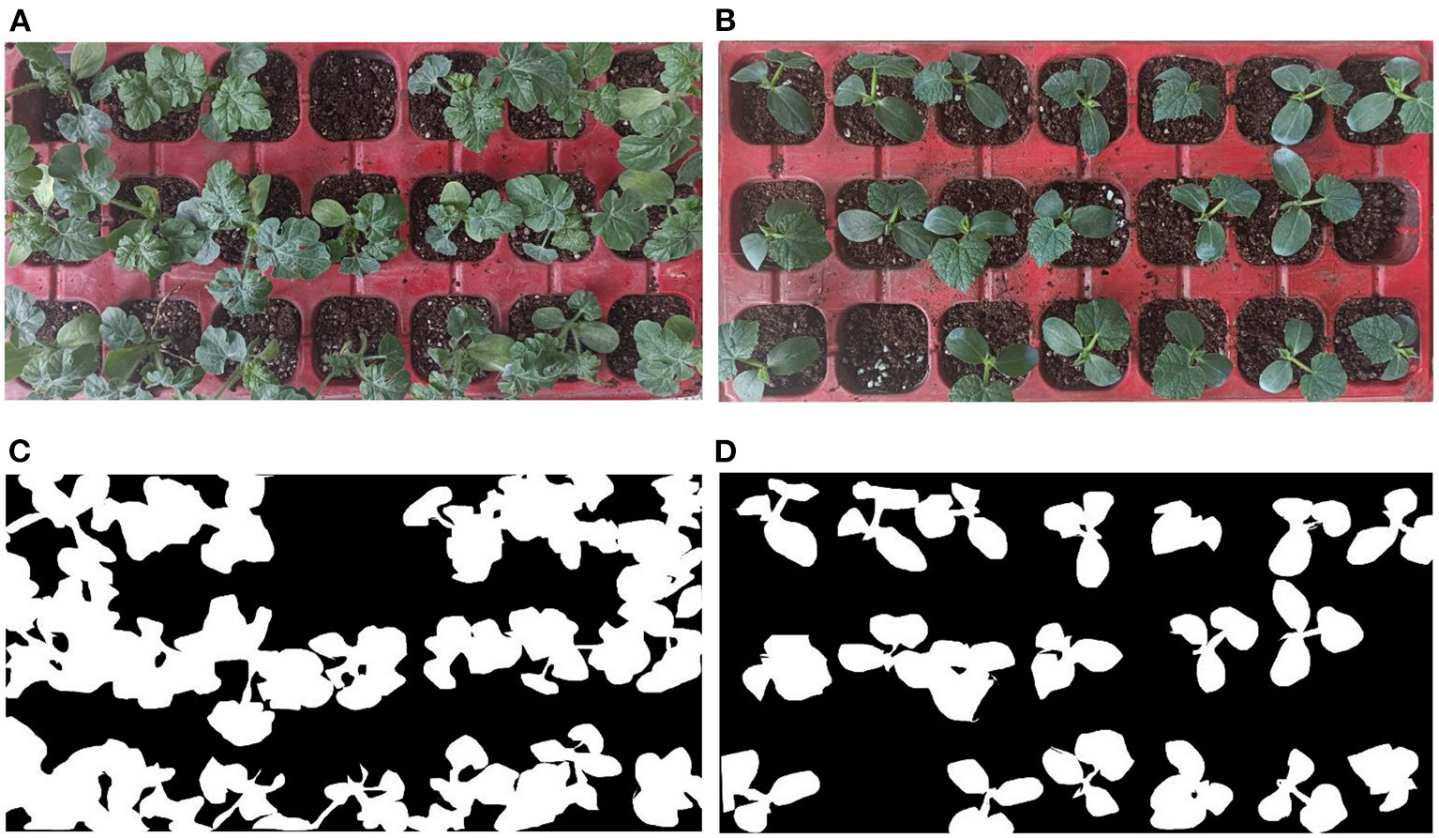
The segmentation results of different species of seedlings. **(A)** Original image of watermelon seedlings, **(B)** original image of cucumber seedlings, **(C)** segmented image of watermelon seedlings, and **(D)** segmented image of cucumber seedlings.

To verify the effectiveness of the proposed method, different species of seedlings were used in the validation experiments of effectiveness. The original pictures of watermelon seedlings and cucumber seedlings are shown in the Figures 7A,B, respectively. The segmentation results of the watermelon seedlings and cucumber seedlings are shown in Figures 7C,D based on the proposed method.

### Identification of Results

#### Threshold Calculation

Twenty groups of pepper seedlings including 2,100 seedlings were analyzed, and the measured threshold “*F*” of each group was calculated. The maximum, average, and minimum values of the pepper seedling thresholds of each group were recorded in Table 1. The average value, *Fa*, of 20 groups was 0.20, the maximum value, *F*_max_, was 0.25, and the minimum value, *F*_min_, was 0.16.

**Table 1:** Threshold statistics of seedings.

**Serial number**	**Threshold F**			**Serial number**	**Threshold F**		
	**Max**	**Average value**	**Minimum value**		**Max**	**Average value**	**Minimum value**
1	0.2	0.18	0.16	11	0.21	0.2	0.19
2	0.25	0.24	0.23	12	0.16	0.16	0.16
3	0.24	0.22	0.2	13	0.17	0.17	0.17
4	0.18	0.17	0.16	14	0.22	0.21	0.2
5	0.18	0.17	0.16	15	0.21	0.19	0.17
6	0.24	0.23	0.22	16	0.25	0.22	0.19
7	0.25	0.24	0.23	17	0.2	0.19	0.18
8	0.16	0.16	0.16	18	0.25	0.25	0.15
9	0.24	0.23	0.22	19	0.23	0.2	0.17
10	0.16	0.16	0.16	20	0.22	0.21	0.2

**Figure 8:**
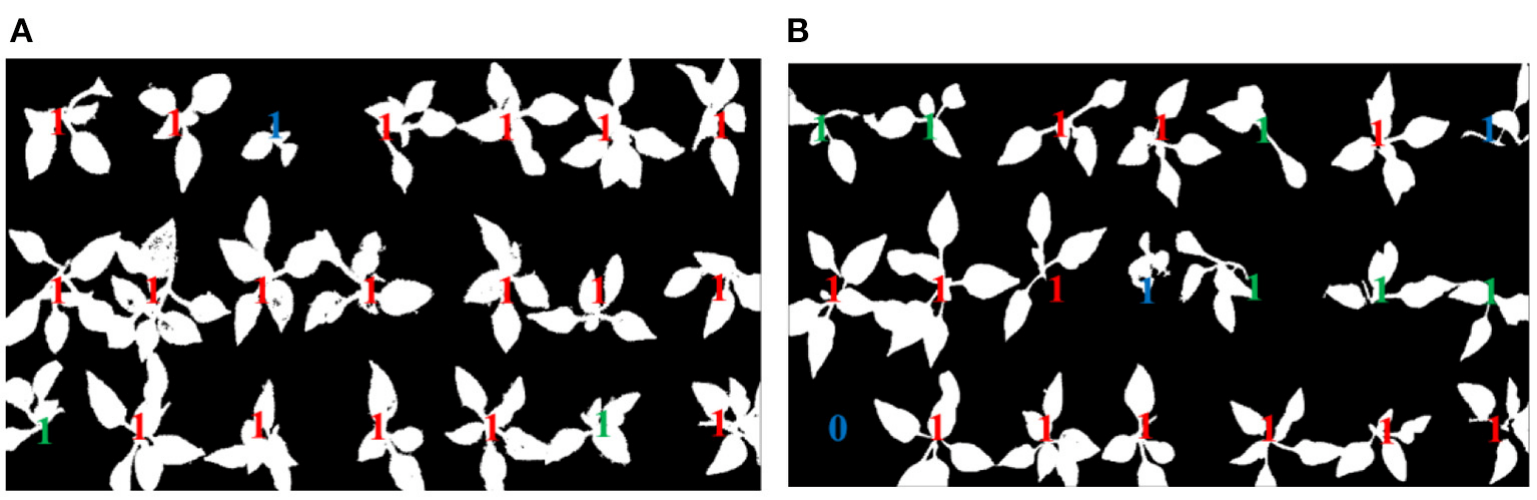
Identification results based on the proposed method.

**Figure 9:**
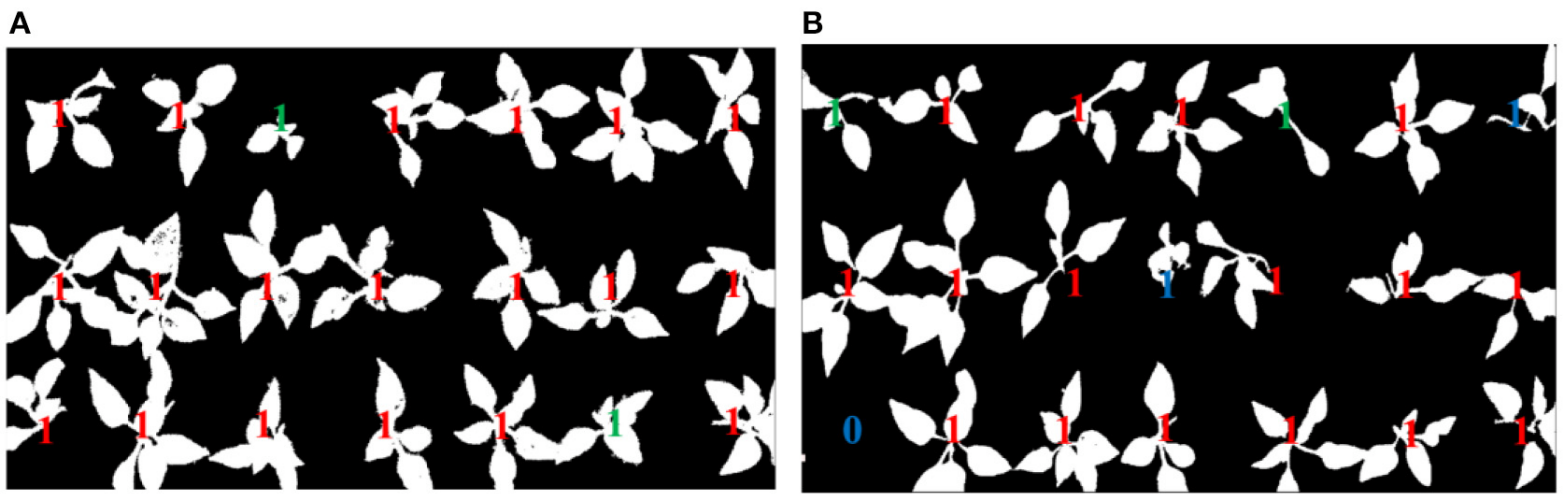
Identification results based on the method in Tong et al. (2013).

The healthy seedling is labeled by using red “1,” the sub-healthy one is labeled by using green “1,” the seedling of poor quality is labeled by using blue “1,” and the empty cell of the tray is labeled by blue “0.” The identification results of original pepper seedling images are shown in Figures 8, 9, which are based on the proposed framework and method in Tong et al. (2013), respectively.

#### Coordinates Determination of Healthy Seedling

**Figure 10:**
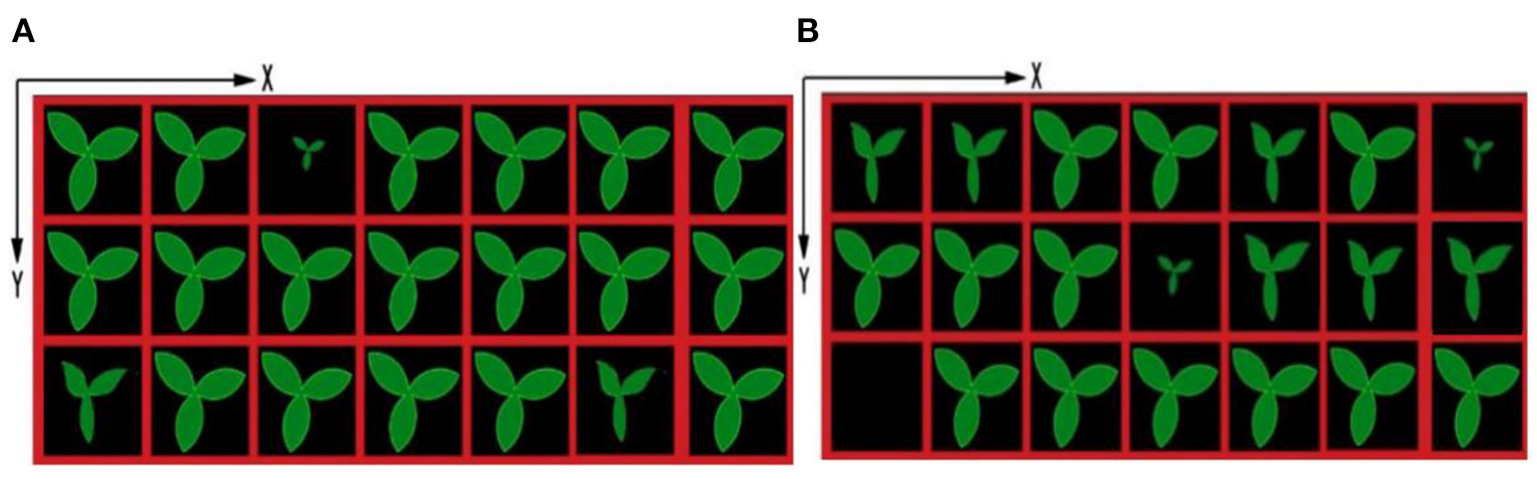
Output ordinates based on the proposed method.

**Figure 11:**
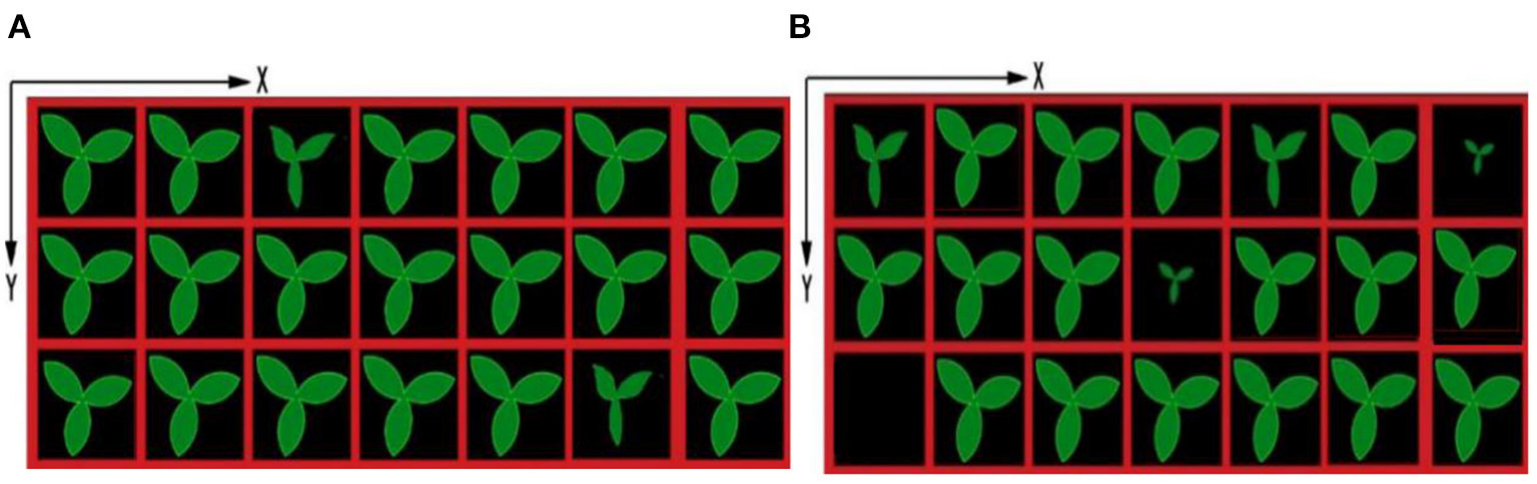
Output ordinates based on the method in Tong et al. (2013).

After identification of the growth state of seedlings, the coordinates of different growth states of seedlings are output shown in Figures 10, 11, which are based on the proposed framework and method in Tong et al. (2013), respectively. The origin of the coordinate system is the upper left corner of the whole tray image. The positive direction of the X-axis of the coordinate system is along the upper edge of the image to the right. The positive direction of the Y-axis of the coordinate system is downward along the left edge of the image. By using the proposed method, the coordinates of sub-healthy seedlings center are (1,5) and (11,5) in Figure 10A and (1,1), (3,1), (9,1), (9,3), (11,3), and (13,3) in Figure 10B, the coordinates of seedlings of poor quality are (5,1) in Figure 10A and (13,1) and (7,3) in Figure 10B, and the coordinates of empty tray cells are (1,5) in Figure 10B. Other coordinates represent healthy seedlings in Figure 10. By comparison, the coordinates of sub-healthy seedlings center are (5,1) and (11,5) in Figure 11A and (1,1) and (9,1) in Figure 11B, the coordinates of seedlings of poor quality are (13,1) and (7,3) in Figure 11B, and the coordinates of empty tray cells are (1,5) in Figure 11B. Other coordinates represent healthy seedlings in Figure 11.

A total of 273 pictures of three groups of seedlings were used to verify the potential of the proposed method. The images were randomly obtained under different seedling growth conditions. There are 75 healthy seedlings, 6 sub-healthy seedlings, 2 poor seedlings, and 1 empty tray cell in group A; 94 healthy seedlings, 6 sub-healthy seedlings, 3 poor seedlings, and 2 empty tray cells in group B; and group C has 72 healthy seedlings, 6 sub-healthy seedlings, 4 poor seedlings, and 2 empty tray cells. The statistics data is recorded in Tables 2, 3. The accuracy rates of healthy seedlings identification are 96, 92.55, and 94.44% using the proposed method in Group A, B, and C, respectively. The accuracy rates of healthy seedlings identification are 92, 87.23, and 88.89% using the comparison method in Group A, B, and C, respectively. The average accuracy rates are 94.33 and 89.37%, which were obtained by using the proposed method and the comparison method, respectively.

**Table 2:** Identification results and accuracy.

	**Healthy seedling**	**Sub-healthy seedling**	**Poor seedling**	**Empty seedling**	**Accuracy (%)**
**GROUP A**					
Group A seedlings	75	6	2	1	100.00
Group A is based on the proposed method	72	4	2	1	96.00
Group A based on the comparison method	69	4	1	1	92.00
**GROUP B**					
Group B seedlings	94	6	3	2	100.00
Group B is based on the proposed method	87	3	1	1	92.55
Group B based on the comparison method	82	4	2	1	87.23
**GROUP C**					
Group C seedlings	72	6	4	2	100.00
Group C is based on the proposed method	68	4	2	2	94.44
Group C based on the comparison method	64	3	3	1	88.89

**Table 3:** Analysis of recognition accuracy under different methods.

	**Accuracy of group A (%)**	**Accuracy of group B (%)**	**Accuracy of group C (%)**	**Average value (%)**
The proposed method	96.00	92.55	94.44	94.33
The comparison method	92.00	87.23	88.89	89.37

## Discussion

It can be seen from the Figures 5, 6 that the proposed method removed the noise effectively and filled the holes on the leaf surface, which also implied that the proposed method was robust against the varied illumination because this experiment was not conducted under the structured light environment. The threshold “*F*” obtained by a lot of experimental analysis could clearly distinguish seedlings of different quality. Although the “*F*” value of the empty tray cell should be 0 in theory, a lot of experiments showed that the value of the tray empty cell was below 0.015. By setting different thresholds “*F*,” different quality seedlings can be accurately distinguished, which can be shown in Figure 7. It can be seen that Figure 7 is all the correct identification results of Figure 4. The proposed method is superior to the comparison method in the identification of healthy seedlings and sub-healthy seedlings, which implies the algorithm can segment and restore seedling leaves effectively. The SIHVS algorithm can use the threshold “*F*” to count the surface pixels of seedling leaves in detail. The average value, *F*_*a*_, is 0.20, the maximum value, *F*_max_, is 0.25, the minimum value, *F*_*min*_ is 0.16, and 0.015 can be set to distinguish the healthy seedlings, sub-healthy seedlings, seedlings of poor quality, and empty tray cells well, which can be confirmed in Tables 2, 3. The proposed method can give a good identification rate of healthy seedlings that is 94.33% and output the coordinates of healthy seedlings for the preparation of transplanting.

## Conclusion

This study proposed a computer vision-based identification framework of healthy seedlings. The healthy seedlings, sub-healthy seedlings, seedlings of poor quality, and empty tray cells could be identified automatically. The BM3D-based method could effectively segment the image of the seedlings by filling the holes on the leaf surface. The SIHVS algorithm could identify the growth status of the seedling by counting the pixel number of the seedling leaf. The threshold “*F*” that is the ratio of the leaf area value of the seedling to the area of empty tray cell is an important index to distinguish seedling growth state. It was found that *F*_*a*_ was 0.20, *F*_max_ was 0.25, *F*_min_ was 0.16, and 0.015 was the boundary values to distinguish the healthy seedlings, sub-healthy seedlings, seedlings of poor quality, and empty tray cells. The proposed method could output the coordinates of healthy seedlings for transplanting grasping. The identification accuracy rate of healthy seedlings was 94.33% higher than 89.37% obtained by the comparison method. The above conclusions imply that the proposed method can identify healthy seedlings in the tray which can be prepared well for transplanting seedlings.

## Data Availability Statement

The original contributions presented in the study are included in the article/supplementary material, further inquiries can be directed to the corresponding author/s.

## Author Contributions

XJ and CW contributed to conception and design of the study. KC and JJ organized the database. SL and YW performed the statistical analysis. XJ wrote the first draft of the manuscript. CW, KC, JJ, and SL wrote sections of the manuscript. All authors contributed to manuscript revision, read, and approved the submitted version.

## Conflict of Interest

The authors declare that the research was conducted in the absence of any commercial or financial relationships that could be construed as a potential conflict of interest.

## Publisher's Note

All claims expressed in this article are solely those of the authors and do not necessarily represent those of their affiliated organizations, or those of the publisher, the editors and the reviewers. Any product that may be evaluated in this article, or claim that may be made by its manufacturer, is not guaranteed or endorsed by the publisher.

## References

[B1] Ashraf M. A. Tian S. Kondo N. (2014). Machine vision to inspect tomato seedlings for grafting robot. Acta Horticult. 1054, 309–316. 10.17660/ActaHortic.2014.1054.37

[B2] Feng Q. Chen J. Lin C. Wang X. (2018). Seedling leaf morphology measurement based on photometric stereo. Trans. Chin. Soc. Agric. Mach. 5, 43–50. 10.1016/j.ifacol.2018.08.109

[B3] Feng Q. Liu X. Jiang K. Fan P. Wang X. (2013). Development and experiment on system for tray-seedling on-line measurement based on line structured-light vision. Trans. Chin. Soc. Agric. Eng. 29, 143–149. 10.3969/j.issn.1002-6819.2013.21.018

[B4] Golbach F. Kootstra G. Damjanovic S. Otten G. Zedde R. (2016). Validation of plant part measurements using a 3d reconstruction method suitable for high-throughput seedling phenotyping. Mach. Vision Appl. 27, 663–680. 10.1007/s00138-015-0727-5

[B5] Han C. Yuan P. Guo H. Zhang J. (2018a). Development of an automatic pepper plug seedling transplanter. Int. J. Agr. Biol. Eng. 27, 110–120.

[B6] Han L. Mao H. Francis K. Hu J. (2018b). Development of a multi-task robotic transplanting workcell for greenhouse seedlings. Appl. Eng. Agric. 34, 335–342. 10.13031/aea.12462

[B7] Hang L. Tang L. Steven W. Mei Y. (2017). A robotic platform for corn seedling morphological traits characterization. Sensors 17:2082. 10.3390/s17092082 PMC5621065 28895892

[B8] Jin X. Cheng Q. Zhao B. Ji J. Li M. (2020). Design and test of 2zym-2 potted vegetable seedlings transplanting machine. Int. J. Agr. Biol. Eng. 13, 101–110. 10.25165/j.ijabe.20201301.5494

[B9] John L. (2017). Machine vision system detects holes in pressed aluminum containers. Vis. Syst. Des. 22:9.

[B10] Karimi S. (2009). Estimation of leaf growth on the basis of measurements of leaf lengths and widths, choosing pistachio seedlings as model. Aust. J. Basic Appl. Sci. 3, 1070–1075.

[B11] Kim W.-S. Lee D.-H. Kim Y.-J. (2020). Machine vision-based automatic disease symptom detection of onion downy mildew. Comput. Electron Agr. 168:105099. 10.1016/j.compag.2019.105099

[B12] Lin T. T. Chien C. F. Liao W. C. (2002). Machine vision approaches for vegetable seedling growth measurement. Acta Hortic. 578, 307–314. 10.17660/ActaHortic.2002.578.38

[B13] Ling P. P. Ruzhitsky V. N. (1996). Machine vision techniques for measuring the canopy of tomato seedling. J. Agric. Eng. Res. 65, 85–95. 10.1006/jaer.1996.0082

[B14] Mauro Z. Veronica R. Fabio L. Malcolm M. Valentino B. Donata C. (2018). Development of a machine vision method for the monitoring of laying hens and detection of multiple nest occupations. Sensors 18:132. 10.3390/s18010132 PMC5796280 29303981

[B15] Rahul K. Raheman H. Paradkar V. (2019). Design and development of a 5r 2dof parallel robot arm for handling paper pot seedlings in a vegetable transplanter. Comput. Electron Agr. 166:105014. 10.1016/j.compag.2019.105014

[B16] Sun L. Chen X. Wu C. Zhang G. Xu Y. (2017). Synthesis and design of rice pot seedling transplanting mechanism based on labeled graph theory. Comput. Electron Agr. 143, 249–261. 10.1016/j.compag.2017.10.021

[B17] Tong J. H. Li J. B. Jiang H. Y. (2013). Machine vision techniques for the evaluation of seedling quality based on leaf area. Biosyst. Eng. 115, 369–379. 10.1016/j.biosystemseng.2013.02.006

[B18] Vivek P. Duraisamy V. M. Kavitha R. (2017). Development of an automatic transplanting mechanism for protray vegetable seedlings, Madras Agric. J. 104, 401–404. 10.29321/MAJ.2017.000087

[B19] Wang C. Lee W. S. Zou X. D. Choi Gan H. Diamond J. (2018). Detection and counting of immature green citrus fruit based on the local binary patterns (LBP) feature using illumination-normalized images. Precis. Agric. 19, 1062–1083. 10.1007/s11119-018-9574-5

[B20] Yang Q. Huang G. Shi X. He M. Ibrar A. Zhao X.. (2020). Design of a control system for a mini-automatic transplanting machine of plug seedling, Comput. Electron Agr. 169:105226. 10.1016/j.compag.2020.105226

[B21] Yang S. Gao W. Jiaqi M. I. Mengliu W. U. Wang M. Zheng L. (2019). Method for measurement of vegetable seedlings height based on RGB-D camera. Trans. Chin. Soc. Agric. Mach. 50, 128–135. 10.6041/j.issn.1000-1298.2019.S0.021

[B22] Zhang L. He H. Wu C. (2015). Vision method for measuring grafted seedling properties of vegetable grafted robot. Trans. Chin. Soc. Agric. Eng. 31, 32–38. 10.11975/j.issn.1002-6819.2015.09.006

